# Improving PHA production in a SBR of coupling PHA-storing microorganism enrichment and PHA accumulation by feed-on-demand control

**DOI:** 10.1186/s13568-018-0628-x

**Published:** 2018-06-12

**Authors:** Shanwen Zeng, Fuzhong Song, Peili Lu, Qiang He, Daijun Zhang

**Affiliations:** 1 State Key Laboratory of Coal Mine Disaster Dynamics and Control, Chongqing University, Chongqing, 400044 China; 2 Department of Environmental Science, Chongqing University, Chongqing, 400044 China; 3 Faculty of Urban Construction and Environmental Engineering, Chongqing University, Chongqing, 400044 China; 4 Key Laboratory of the Three Gorges Reservoir’s Eco-Environments, Ministry of Education, Chongqing University, Chongqing, 400044 China

**Keywords:** Poly-β-hydroxybutyrate (PHB), Mixed culture, Coupling, Settling selection, Feed-on-demand control

## Abstract

With volatile fatty acids as substrates, the typical polyhydroxyalkanoates (PHAs) production by mixed culture always includes two steps: PHA-storing culture enrichment via aerobic dynamic feeding strategy and PHA accumulation under nutrient-limited condition. To simplify the PHA-production steps, the enrichment and accumulation step were coupled in a SBR. At start-up period, to investigate the effect of settling selection, one acetate-fed SBR was operated by settling selection-double growth limitation (SS-DGL) strategy, while the other was operated by DGL strategy. The results showed that the stable operation in SBR1 was obtained at about 21, 12 days faster than SBR2, implying the settling selection accelerated the start-up process. After omitting the settling selection under the stable operation, the SBR1 was run above 15 days. The results showed that the performance was not substantial altered. Therefore, the settling selection affected the start-up process but not the stable operation. At operational period, based on the sharp decreasing of oxygen uptake rate (OUR), the poly-β-hydroxybutyrate (PHB) content was improved 13%, from 70 to 83% by feed-on-demand control-double growth limitation (FD-DGL). And the harvested volumetric productivity was 5.0 gPHB/L/day, almost 1-folder improvement. That was to say, the PHB production in a SBR of coupling the enrichment and accumulation step was improved by feed-on-demand control. Meanwhile, the FD experiment can keep steady running for 10 SRTs. Therefore, the SS-DGL/FD-DGL strategy was a promising method for PHA production.

**Electronic supplementary material:**

The online version of this article (10.1186/s13568-018-0628-x) contains supplementary material, which is available to authorized users.

## Introduction

The widely use of conventional plastics in daily life causes unfriendly effect on our environment, due to those low biological degradation, incompatibility with the environment and non-renewability (Fradinho et al. 2014; Khanna and Srivastava 2005). Therefore, a type of environmentally friendly new materials, possessing similar physicochemical characteristics to conventional plastics, are urgent needed. Polyhydroxyalkanoates (PHAs) are microbial storage polymers, served as an intracellular carbon and energy reserve (Tan et al. 2014). The physicochemical properties of PHAs are similar to the conventional plastics, but biodegradable and renewable (Kourmentza and Kornaros 2016; Laycocka et al. 2014; Prieto et al. 2016; Reddy et al. 2017), which attracted more and more attention of researchers.

To reduce the cost of PHA production and allow broad application, mixed culture instead of pure culture was usually used to product PHA (Salehizadeh and Van Loosdrecht 2004; Serafim et al. 2008a). The typical process of PHA production includes three steps: acidogenic fermentation, enrichment of PHA-storing culture and PHA accumulation (Amulya et al. 2015; Dionisi et al. 2004; Serafim et al. 2008a). The first step is to convert wastes and wastewaters to volatile fatty acids (VFAs). These short chain acids are more suitable for PHA production and will be used in the following two steps. The aiming of the second step includes: (1) to enrich bacterium capable of PHA storing, (2) to sustain high growth rate of PHA-storing culture to supply the PHA accumulation step (Oliveira et al. 2017). The enrichment of PHA-storing culture is carried out by aerobic dynamic feeding (ADF) strategy (Johnson et al. 2009). The simultaneous feeding of carbon sources and nutrients in the initial period of feast phase (external substrates are sufficient) led to shorter feast phase and longer famine phase (external substrates are insufficient) (Reis et al. 2003). Non-storing bacterium incapable of storing cannot bear longer starvation thus would be outcompeted (Johnson et al. 2009). Marang et al. (2016) fed the carbon source and nutrients separately, carbon source fed in the initial state of the feast phase and nutrients fed in the initial state of the famine phase. This new strategy was called double growth limitation (DGL) strategy, and the conventional ADF strategy was called single growth limitation strategy by Oliveira et al. (2017) and Silva et al. (2017).

Differing from the mechanism of ecological selection, physical selection also can be applied to enrich PHA-storing bacterium (Chen et al. 2015). Considering that after storage process that cell destiny of PHA-storing bacterium exceeds the one of non-PHA-storing bacterium, the strategy of aerobic dynamic discharge (ADD), adding a fast setting and withdrawal process at the end of feast phase, was used to enrich the PHA-storing bacterium (Chen et al. 2015). However, the enrichment process based on the combination of the two mechanisms was not reported. Finally, to achieve the maximum PHA content, the third or PHA accumulation step was performed under nutrient-limited condition by the bacterium enriched in the second step. However, some researchers considered that the PHA-rich sludge at the end of feast phase from the enrichment step can directly harvest PHA (Dionisi et al. 2007; Reis et al. 2003). Marang et al. (2016) also considered that the accumulation step increased the cost of PHA production.

Previously, most of researches were focused on the use of cheaper raw materials (Solaiman et al. 2006; Sudesh et al. 2011), improvement of PHA-storing bacterium abundance (Salehizadeh and Van Loosdrecht 2004) and PHA production extension (Huang et al. 2017). The study about the coupling of enrichment step and accumulation step was scarcely reported. Marang et al. (2016) showed that the maximum PHA content in the SBR of the coupling was 70%. However, the result was less than the one (86%) in fed-batch reactor for accumulation experiments (Marang et al. 2016) and the one (89%) reported by Johnson et al. (2009), implying that the PHA production can be improved. Continuous and pulsed feeding are the two common methods for substrate feeding in PHA production process. Although minority of reports considered that continuous feeding was superior to pulsed feeding (Albuquerque et al. 2011), pulsed feeding was more suitable for PHA production (Johnson et al. 2009; Serafim et al. 2004). By contrasting substrate uptake rate, PHA storage rate and PHA storage content under different feeding regimens, the results showed that three pulses was superior to single pulsed and continuous feeding (Serafim et al. 2004, 2008b). And pulsed feeding was usually applied to PHA accumulation by researchers, such as, 5 pulses (Chen et al. 2016), 15 pulses (Campanari et al. 2017), continuous pulsed feeding (Chen et al. 2013), feed-on-demand control (Johnson et al. 2009; Marang et al. 2016; Valentino et al. 2015). Pulsed feeding strongly improved the PHA storage efficiency in cell due to avoiding the effect of substrate inhibition (Albuquerque et al. 2007; Serafim et al. 2004). Nevertheless, the study about applying feed-on-demand control to the coupling lacked of reports.

The aiming of this study is to improve the PHA production in a SBR of coupling enrichment and accumulation by feed-on-demand control. The main research included: (1) the effect of settling selection on the start-up process; (2) whether to be able to achieve the stable coupling and improve the PHA production by the application of feed-on-demand control; (3) discuss the feasibility of coupling enrichment and accumulation step.

## Materials and methods

### Sequencing batch reactor for the coupling at start-up period

At start-up period, two SBRs were set up to launch the coupling and investigate the effect of settling selection (SS). Both of the SBRs were performed by double growth limitation (DGL) strategy via separate carbon-feeding and nutrients-feeding. The difference between the two SBRs was whether to a settling selection was applied (SBR1) or not (SBR2). Initial sludge inoculated from the aeration tank of a waste water treatment plant (WWTP) in Chongqing, China. The height-diameter ratio of the two SBRs is 10:1. And the working volume is about 3.4 L. Both of the SBRs were operated with 18 h cycles. SBR1 cycle (18 h) consisted of 360 min feast phase (including carbon-feeding during the initial 15 min, 3/4 mixture withdrawn by battery valve at the last 3 min), 25 min settling selection (including 12 min inflow of water, 10 min settling and 3 min effluent by battery valve) and 695 min famine phase (including nutrients-feeding during the initial 15 min) (Additional file 1: Fig. S1a), where, the settling time was referred to Chen et al. (2015). SBR2 cycle (18 h) consisted of 360 min feast phase (including carbon-feeding during the initial 15 min, 3/4 mixture withdrawn by battery valve at the last 3 min) and 720 min famine phase (including nutrients-feeding during the initial 15 min). SBR1 was set up with a settling phase, correspondingly, SBR2 was set up without a settling phase. Therefore, sludge retention time (SRT) in SBR1 was slightly less than 1 day and SRT in SBR2 was 1 day.

The dosage of carbon- and nutrients-feeding was performed according to Marang et al. (2016). During the period of carbon-feeding, 250 mL 0.85 M sodium acetate solution was fed into the two SBRs. Because acetate was the sole substrate, the stored production was only PHB (Jiang et al. 2012). The dosage volume of nutrients solution was 2.3 L, including solution A 50 mL, solution B 2 mL/L, ATU 5.5 mg/L (to prevent nitrification) and water approximately 2.25 L. Solution A (1 L) consisted of 17.905 g (NH_4_)_2_SO_4_, 17.522 g KH_2_PO_4_, 7.115 g MgSO_4_∙7H_2_O, 2.776 g KCl. And solution B (1 L) comprised 0.12 g ZnSO_4_∙7H_2_O, 0.15 g CaCl_2_∙7H_2_O, 0.12 g MnCl_2_∙4H_2_O, 0.06 g Na_2_MoO_4_∙2H_2_O, 0.03 g CuSO_4_∙5H_2_O, 0.15 g CoCl_2_∙6H_2_O, 3.0 g EDTA, 1.5 g FeCl_3_∙7H_2_O and 0.15 g H_3_BO_3_.

Dissolved oxygen (DO) supplied by air pump was above 2 mg/L. The temperature in the reactor controlled at about 30 °C by water bath. PH was not controlled and varied from 8 to 9 (Chen et al. 2015; Serafim et al. 2004), which was suitable for PHA-storing culture selection (Chen et al. 2013; Chua et al. 2003).

In addition, to investigate the effect of omitting the settling selection under the stable operation, the settling selection of SBR1 was omitted. The conditions of temperature, pH, DO and nutrients were same to SBR1. Under this new condition, the SBR was run above 15 days.

### Batch experiment for PHB storage capacity

A batch experiment was setup to estimate the maximum PHB storage capacity of the culture. The accumulation experiments were carried out in a respirometer. At the beginning of each experiment, the 4 L respirometer was filled with the sludge from the SBR, and carbon- and ammonium-free medium (otherwise the same composition). If no acetate was detected, the batch experiment was activated by pulsed feeding 60 mmol sodium acetate. Further carbon source was continuously fed into the respirometer via dOUR/dt signal (see the following description), using a 1.5 M sodium acetate solution. Microbial growth was prevented throughout the experiment, due to no ammonium feeding.

### Description for feed-on-demand control

During PHA production process, PHA concentration cannot be measured online, therefore, the storage process was usually controlled by indirect method. The signal of dissolved oxygen (DO) is commonly used to direct the end of PHA storage, and the steep rise of DO signal indicates the exhaustion of substrate and the maximum PHA content (Valentino et al. 2015; Werker et al. 2014). The sharp drop of oxygen uptake rate (OUR) also indicates the end of storage (Serafim et al. 2004). This means that online OUR signal can also be used to direct the consumption of substrate and the storage of PHA. Based on this cognition, this study applied feed-on-demand control to direct the substrate feeding according to online OUR date. Online OUR monitoring was carried out on a Labview platform, details referencing to Lu et al. (2006). A liquid-flowing cycle occurs between a 3 L reactor and a 1 L reactor, the bigger one is an aeration tank and the smaller one is a reaction tank. Two dissolved oxygen (DO) sensors were applied to monitoring the inflow and the outflow DO of the reaction tank. And OUR is calculated by the difference between the two DO values divide time. The order was performed according to online OUR date. When dOUR/dt < 0, micro pump started to work to feed carbon source, in reverse, when dOUR/dt ≥ 0, micro pump stopped. The details are as follows:

i. Stable OUR level was obtained before pulsed feeding. Stable OUR level means that initial background or endogenous respiration rate for the biomass, and the initial PHA content is negligible;
ii. The first pulsed feeding launched in accordance with a compulsive START order from Labview platform. Subsequent pulsed feeding was carried out based on the actual monitoring of oxygen consumption of biomass;
iii. Continuous pulsed feeding was determined by the OUR signal. Pulsed feeding worked, when the signal of OUR had a relative decline.
  $$ \frac{{OUR_{n + 1} - OUR_{n} }}{nt} < 0 $$
  where, OURn was the OUR value of the nth (n ≥ 1) sampling point; OURn + 1 was the OUR value of the (n + 1)th (n ≥ 1) sampling point; ∆t was the time interval between two neighbouring OUR signal, 0.5 min.
iv. To guarantee the SBR stable running, the total fed substrate amount was controlled. When the fixed amount of substrate was exhausted, OUR was due to sustain continuous endogenous respiration, implying biomass being in famine state.

### Feed-on-demand control experiment to improve PHB production at operational period

The feed-on-demand control (FD) experiment was carried out to investigate if it is possible to sustain the SBR steady running and improve the PHB production after the application of feed-on-demand control. The SBR was connected with the reactor of the respirometer. The conditions of pH, temperature, SRT, aeration were same as SBR experiment. The SBR of the coupling was still performed by double growth limitation (DGL) strategy (Additional file 1: Fig. S1b). The feeding regimen was just changed to feed-on-demand control based on the signal of dOUR/dt, details see the above paragraph. Meanwhile, to maintain the system stable, the total amount of substrate fed in FD experiment was controlled to keep the same feast length to SBR experiment. Pulsed concentration was referred to Johnson et al. (2009) and Marang et al. (2016). The FD experiment was run for 10 SRTs (10 days) under this condition.

### Analytical methods

Mixed liquor suspended solids (MLSS), mixed liquor volatile suspended solids (MLVSS) and ammonium (NH4+-N) were analyzed according to the standard methods for the examination of water and wastewater (APHA 1998).

Acetic acid (HAc) was quantified by gas chromatography (GC) according to Yuan et al. (2006). The pH of filtered samples (0.45 µm) were adjusted to about 4 by 3% (v/v) H_3_PO_4_. 1 µL sample was used to test HAc by GC (GC-2010plus, Shimadzu) with a flame ionization detector (FID) and a capillary column (DB-FFAP, Agilent, 30 m * 0.25 µm * 0.25 mm).

PHAs were quantified by gas chromatography (GC) using a method adapted from (Oehmen et al. 2005). Samples collected in 10 mL threaded tube were centrifuged for 10 min at 4500 rpm. After removing the supernatant, the samples were freeze-dried for 24 h in a vacuum freeze drier (SJIA-10N, Ningbo Shuangjia, China) to weigh the TSS. After adding 2 mL chloroform and 2 mL acidified methanol solution (10% H_2_SO_4_, including approximately 100 mg/L sodium benzoate as an internal standard), the lyophilised biomass was digested at 100 °C for 6 h. The samples were cooled to room temperature and then 1 mL distilled water was added in. After mixing, the bottom phase was then transferred to a vial. Finally, 1 µL sample was injected into GC (GC-2010plus, Shimadzu) with a flame ionization detector (FID) and a capillary column (RTX-1, 30 m * 0.25 µm * 0.25 mm). The overall amount of stored polymer was quantified by using a mixed standard with HB: HV 90 to 10% (w/w) (Sigma-Aldrich, US).

The 50 mL SBR1 and SBR2 samples were taken from SBR1 and SBR2 under stable operation to quantify the abundance by high throughput sequencing. All samples were immediately stored at − 20 °C and would be used in the following test. According to manufacturer’s instructions, the total genomic DNA from samples was extracted in triplicate using the PowerSoil DNA Isolation Kit (MoBio, Carlsbad, CA). The V3, V4 region of the 16S rDNA gene was amplified using bacterial primers 27F AGAGTTTGATCCTGGCTCAG and 534R ATTACCGCGGCTGCTGG (Wang et al. 2017). The amplicons were purified using MiSeq Reagent Kit v3 (MS-102-3003) (Illumina, USA). Ineffective reads (ambiguous nucleotides and quality value < 20) were removed from the raw sequence data. And effective sequences were clustered into operational taxonomic units (OTUs) at 97% sequence identity, then the highest of each OTU was selected as the representative sequences. The resulting OTUs were classified following the Greengene database (DeSantis et al. 2006). Finally, the phylogenetic tree was constructed using MEGAN. The Illumina sequencing raw data was deposited as a NCBI BioProject (Accession Number PRJNA436073).

### Calculations

Biomass concentration (X) was estimated by volatile suspended solids concentration (VSS) minus PHB, X = VSS − PHA. PHB (mg/L) = VSS × PHB (%). The yields of PHB production per substrate consumption (Y_P/S_) were determined by dividing the maximum PHB production rates (q_P,max_, Cmol/(Cmol h)) by maximum substrate uptake rates (q_Ac,max_, Cmol/(Cmol h)), Y_P/S_ = q_P,max_/q_Ac,max_. The specific PHB synthesis rate (q_P_, Cmol/(Cmol h)) was calculated by dividing the amount of PHB produced in the feast period (PHB, Cmmol) by the active biomass present in the reactor (X, Cmmol) and the duration of the feast period (t, h), q_P_ = PHB/X/t. The specific acetate consumption rate (q_AC_, Cmol/(Cmol h)) was calculated by dividing the amount of acetate consumed in the feast period (Ac, Cmmol) by the active biomass present in the reactor (X, Cmmol) and the duration of the feast period (t, h), q_AC_ = Ac/X/t. The volumetric productivity (g PHB/L/h) was calculated by dividing the stored PHB by the working volume and reaction time (Huang et al. 2017).

The active biomass concentration was converted from g/L into carbon moles per liter (Cmol/L) according to its monomer formula, CH_1.8_O_0.5_N_0.2_, with a molecular weight of 24.6 g/Cmol (Beuna et al. 2002). PHB concentration was also converted from g/L into Cmol/L under the assumption that PHB had a general composition of C_4_H_6_O_2_ with a molecular weight of 21.5 g/Cmol (Marang et al. 2016).

## Results

### Effect of settling selection

**Fig. 1:**
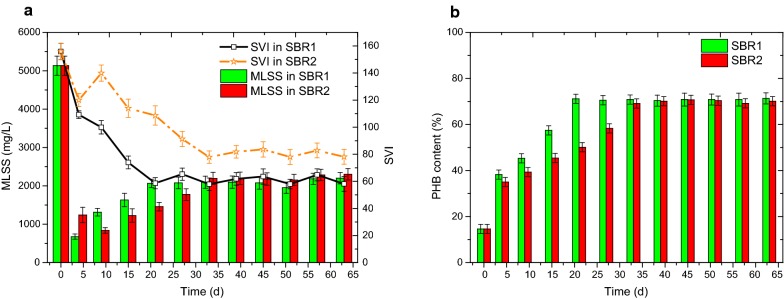
**a** MLSS concentration, SVI and **b** PHB % profiles in SBR1 and SBR2 throughout the experiment

In Fig. 1a, the initial MLSS was 5130 ± 250 mg/L, rapidly decreased to the minimum 680 ± 69 mg/L in SBR1 after 4 days and 840 ± 73 mg/L after 9 days in SBR2. Then, MLSS increased to about 2200 mg/L after 21 days in SBR1 and 2300 mg/L after 33 days in SBR2. SVI decreased from 155 to 60 and 80 in SBR1 and SBR2, respectively. Figure 1b showed PHB_max_% were 71.2 ± 2.2 and 70.1 ± 2.9%, respectively. Obviously, no matter whether to add the settling selection, the maximum PHB content of the two SBRs in the stable phase were both above 70%. However, the stable phase in SBR1 was obtained in 21, 12 days less than SBR2, implying the start-up process in SBR1 was faster, due to the settling selection. The result indicated that adding a settling selection accelerated the start-up process, which is consistent with (Chen et al. 2015). In addition, by high-throughput sequencing, the relative abundance of PHA-storing microorganisms in steady state was 66.4 and 66.7% in SBR1 and SBR2, respectively (Table 1), close to 68.6% (Wang et al. 2017). In Table 1, *Plasticicumulans* (Johnson et al. 2009; Marang et al. 2013), *Azoarcus* (Lemos et al. 2008; Silva et al. 2007), *Zoogloea* (Jiang et al. 2011), *Paracoccus* (Albuquerque et al. 2013) and *Brevundimonas* (Lemos et al. 2008; Silva et al. 2007) have previously been indicated to store PHA. And the types of PHA-storing microorganisms in both SBRs are the same. The same PHA storing performance under stable operation in the two SBRs was verified by the results of the high-throughput sequencing.

**Table 1:** The 12 most abundant bacteria identified by high throughput sequencing in the two SBRs under stable operation

	SBR1			SBR2		
	Phylum	Genus	%	Phylum	Genus	%
1	*Proteobacteria*	***Plasticicumulans***	31.9	*Proteobacteria*	***Plasticicumulans***	23.8
2	*Proteobacteria*	***Azoarcus***	21.3	*Proteobacteria*	***Azoarcus***	21.5
3	*Proteobacteria*	***Zoogloea***	9.8	*Proteobacteria*	***Zoogloea***	19.4
4	*Bacteroidetes*	*Flavobacterium*	3.0	*Bacteroidetes*	*Flavobacterium*	3.5
5	*Proteobacteria*	*Rhizobium*	0.5	*Gammaproteobacteria*	*Acinetobacter*	5.2
6	*Proteobacteria*	*Pseudomonas*	0.1	*Proteobacteria*	*Pseudomonas*	0.7
7	*Proteobacteria*	***Paracoccus***	2.7	*Proteobacteria*	***Paracoccus***	1.3
8	*Proteobacteria*	*Acidovorax*	1.4	*Proteobacteria*	*Acidovorax*	0.9
9	*Proteobacteria*	***Brevundimonas***	0.7	*Proteobacteria*	***Brevundimonas***	0.7
10	*Firmicutes*	*Fusibacter*	0.2	*Firmicutes*	*Fusibacter*	0.6
11	*Verrucomicrobia*	*Prosthecobacter*	2.7	*Verrucomicrobia*	*Prosthecobacter*	0.4
12	*Proteobacteria*	*Uliginosibacterium*	1.3	*Proteobacteria*	*Uliginosibacterium*	0.3
Total reported PHA-storing bacteria (in bold)			66.4	Total reported PHA-storing bacteria (in bold)		66.7

**Fig. 2:**
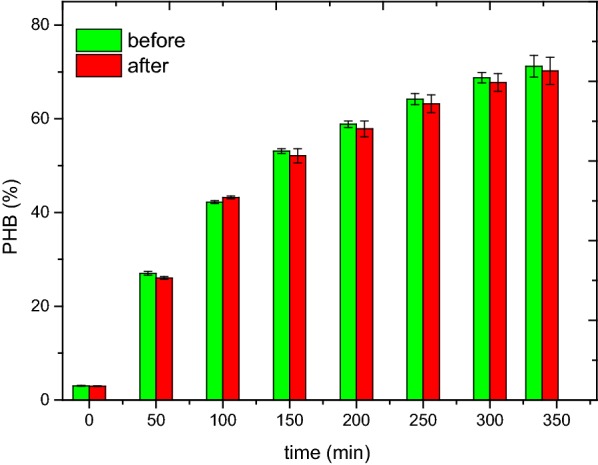
PHB content during the feast phase in a typical cycle before (green) and after (red) omitting the settling selection

After omitting the settling selection under the stable operation, the SBR was run more than 15 days. The reactor performance still remained stable. Figure 2 showed that the PHB content in biomass during the feast phase in a typical cycle before and after omitting the settling selection were comparable. The result showed that PHB content just decreased less than 1%, indicating that omitting the settling selection had a negligible effect on PHB accumulation. As a result, the settling selection affected the start-up process but not the stable operation. The maximum content of PHB was about 70%, still less than the one (86%) in batch experiment (see Table 2). Therefore, other methods to improve PHB production were needed.

**Table 2:** Overview of PHB accumulating performance in SBR, FD and batch experiment

	Time PHB max. (h)	PHB max. (wt%)	Y_P/S_ (CmolPHB/CmolAc)	q_AC_ (CmolAc/CmolX/h)	q_P_ (CmolPHB/CmolX/h)
SBR experiment^a^	5.6 ± 0.3	70.2 ± 1.9	0.60 ± 0.02	0.71 ± 0.04	0.43 ± 0.06
FD experiment^a^	5.6 ± 0.25	83.0 ± 0.8	0.81 ± 0.02	1.05 ± 0.05	0.85 ± 0.07
Batch experiment	Almost 10	86.4 ± 1.2	0.77 ± 0.01	0.83 ± 0.05	0.64 ± 0.06
Johnson et al. (2009)^b^	7.6	89	0.6	NA	NA
Chen et al. (2015)^b^	6.5	74.16 ± 0.03	0.77 ± 0.05	0.82 ± 0.03	0.63 ± 0.06
Marang et al. (2016)^b^	10–12	86 ± 1	NA	NA	NA

^a^ To keep the same feast length, the amount of Ac was 124.5 and 182 Cmmol/L in SBR and FD experiment, respectively

^b^ Date from the accumulation experiments of the three references

### Improving PHB production by feed-on-demand control

**Fig. 3:**
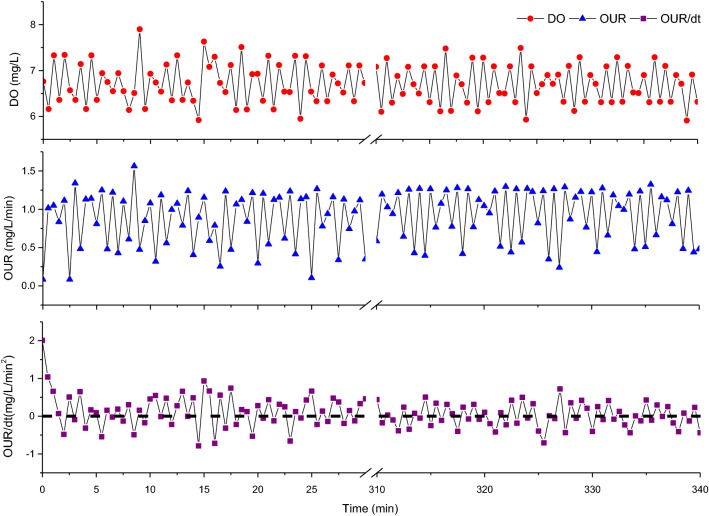
The signal of DO (red cycle), OUR (blue triangle) and dOUR/dt (purple square) during the feast phase in a typical cycle applying the feed-on-demand control

The feed-on-demand control (FD) experiment was setup to investigate whether it is possible to achieve the stable coupling and improve the PHB content. The signal of DO, OUR and dOUR/dt were showed in Fig. 3. The first pulsed feeding launched in accordance with a compulsive START order from Labview platform. Subsequent pulsed feeding carried out based on the actual monitoring of oxygen consumption of biomass. The trend of DO signal was opposite to the signal of OUR. When the curve of DO was rising, the curve of OUR was declining. Vice versa. As the value of dOUR/dt was negative, the substrate was added by micro pump. After adding, the signal of OUR rapidly went up then went down, thus the multiple pulsed signal was generated.

**Fig. 4:**
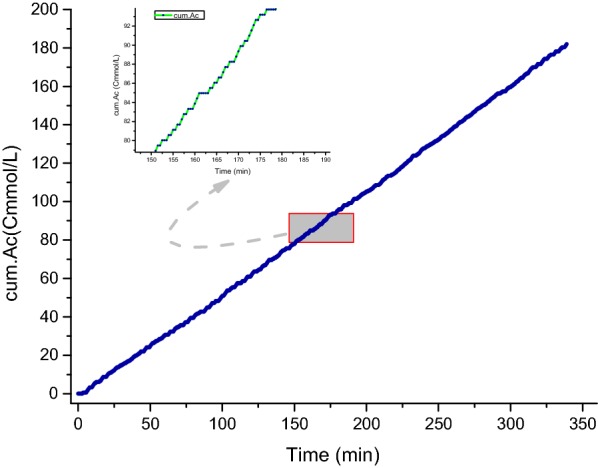
The accumulative amount of HAc fed during the feast phase in a typical cycle applying the feed-on-demand control

To assure the same feast length to SBR and thus maintain the system steady, the total amount of NaAc fed in FD experiment was controlled. 182 Cmmol/L Ac was fed into the SBR in a cycle. In Fig. 4, as a whole, the accumulative Ac presented a rising line, due to the low and constant concentration of pulsed substrate. By zooming in on this figure, the curve presented multistage rising and steady. When dOUR/dt < 0, the substrate was added by micro pump, the curve presented rising. When dOUR/dt ≥ 0, the micro pump stopped working, the curve stayed steady. The rising curve indicated the insufficient substrate in SBR, while the steady one indicated the sufficient substrate in SBR. And the tendency of substrate was agree with the signal of OUR.

**Fig. 5:**
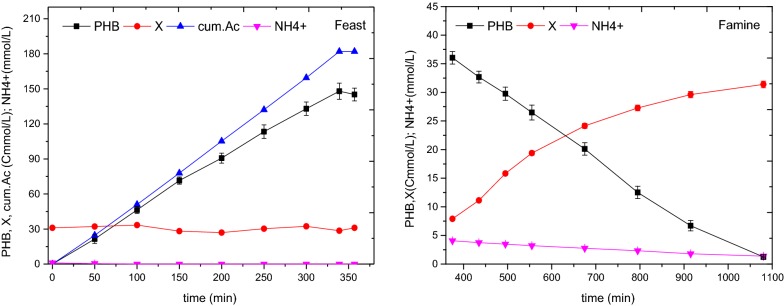
Results in a typical cycle applying the feed-on-demand control, feast phase (left) and famine phase (right)

In Fig. 5, the results of PHB, X and NH4+-N in FD experiment were shown. In feast phase, PHB concentration reached the maximum 148 Cmmol/L, twofold higher than the one in SBR, at about 340 min. And the harvested volumetric productivity was 5.0 gPHB/L/day, twice to the SBR but less than 6.09 gPHB/L/day reported by Oliveira et al. (2017). The smaller volumetric productivity was due to lower biomass in the coupling. The concentration of X was essentially unchanged, about 31 Cmmol/L. A small amount of NH4+-N (about 0.1 mmol/L) was exhausted at a sampling interval (50 min). The specific acetate consumption rate (q_AC_) was 1.05 ± 0.05 Cmol/Cmol/h (Table 2), higher than 0.82 ± 0.03 Cmol/Cmol/h reported by Chen et al. (2015). The specific PHB synthesis rate was 0.85 ± 0.07 Cmol/Cmol/h (Table 2) also higher than 0.63 ± 0.06 Cmol/Cmol/h reported by Chen et al. (2015). The yields of PHB production per substrate consumption (Y_P/S_) was 0.81 ± 0.02 Cmol/Cmol (Table 2) higher than 0.77 ± 0.05 Cmol/Cmol (Chen et al. 2015) and 0.60 Cmol/Cmol (Johnson et al. 2009). At the end of feast phase, 3/4 mixture was withdrawn, biomass was in famine phase. The withdrawn (3/4 volume) sludge was to recover the production of PHB, while the remaining (1/4 volume) sludge supported the growth. At initial famine phase, nutrients were fed into the SBR to allow the growth. NH4+-N decreased from 4.7 mmol/L to about 0.1 mmol/L. Meanwhile, the biomass was in growth process, increasing from about 7.9 to 31 Cmmol/L. The energy in this process was supplied by the PHB stored in feast phase. And PHB degraded about 12 h from about 36 Cmmol/L to nearly zero. From the view of biomass balance, the increasing (growth) was the equivalent of the consumption (withdrawal). The continuous PHA harvest and biomass growth can come true in a SBR.

### PHB accumulating performance in SBR, FD and batch experiment

**Fig. 6:**
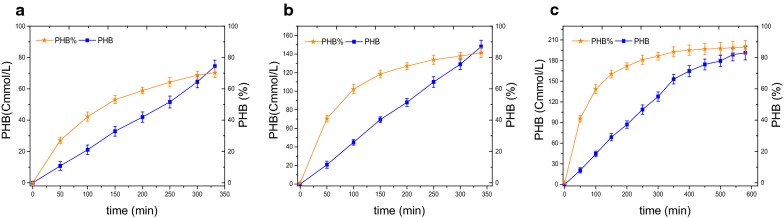
PHB accumulating performance in SBR (**a**), FD (**b**) and batch (**c**) experiment

In Fig. 6, the curve of PHB presented continuous increasing. And the curve of PHB% firstly rose then gradually went steady. The maximum PHB concentration was 191 ± 4 Cmmol/L in batch experiment, more than 148 ± 7 Cmmol/L in FD experiment and 74 ± 5 Cmmol/L in SBR. In SBR, the maximum PHB content was 70.2 ± 1.9%, less than the other two. In batch experiment, the maximum PHB content was 86%, higher than the one (83%) in FD experiment. However, the storage length for reaching the maximum PHB content was near 10 h, longer than the one (about 5.6 h) in SBR and FD experiment. In Fig. 6c, the slope of PHB decreased after 6 h in batch experiment, implying the descent of PHB storage rate. This is similar to the observation of Johnson et al. (2009), once the PHB contents was higher than the one in the SBR cycle, the biomass specific PHB production rate of the culture decreased with the increasing of PHB content.

The length of feast phase of the SBR and FD experiment were both about 5.6 h. And the amount of HAc fed in two experiments were 124.5 and 182 Cmmol/L, respectively. In the same feast length, substrate fed by feed-on-demand control was about 1.5 times higher, implying higher substrate uptake rate. The observation was similar to the result of Serafim et al. (2004), pulsed feeding spent only half time to uptake the same concentration of substrate. The observation was also close to the one of Canelhas et al. (2017), the rate of substrate uptake by pulsed feeding was twice times than the continuous feeding. After the application of feed-on-demand control, the PHB content was improved about 13%, from 70 to 83%. From the view of carbon conversion, in Table 2, the yield of PHB production per substrate consumption (Y_P/S_) in SBR experiment was 0.60 ± 0.02 CmolPHB/CmolAc, close to the one by Johnson et al. (2009). Y_P/S_ in FD experiment was 0.81 ± 0.02 CmolPHB/CmolAc higher than the result of SBR. The specific acetate consumption rate (q_AC_) in FD experiment was 1.05 ± 0.05 CmolAc/CmolX/h higher than 0.71 ± 0.04 Cmol Ac/CmolX/h in SBR. And the specific PHB synthesis rate (q_P_) was 0.85 ± 0.07 CmolPHB/CmolX/h also higher than 0.43 ± 0.06 CmolPHB/CmolX/h in SBR. The feed-on-demand control was hence advantageous to substrate uptake and storage process, indirectly verifying that the effect of substrate inhibition on PHB storage.

The feast length of this study in batch experiment was almost 10 h for reaching the maximum PHB content. Compared to the references in Table 2, the length was 7.6 h (enrichment cycle 12 h) (Johnson et al. 2009), 6.5 h (enrichment cycle 6 h) (Chen et al. 2015) and 10–12 h (enrichment cycle 18 h) (Marang et al. 2016). Obviously, the accumulation step independent of the enrichment step spent more time to reach the maximum PHB content. Although the FD experiment sacrificed about 3% PHB, the coupling of enrichment and accumulation step came true and shorter time (5.6 h) was spent to reach the maximum PHB content (83%). From the view of carbon conversion, the yields of PHB production per substrate consumption (Y_P/S_) in FD experiment was 0.81 ± 0.02 CmolPHB/CmolAc higher than the batch experiment (0.77 ± 0.01 CmolPHB/CmolAc). In addition, the specific PHB synthesis rate (q_P_) was 0.85 ± 0.07 CmolPHB/CmolX/h in FD experiment and 0.64 ± 0.06 CmolPHB/CmolX/h in batch experiment, respectively. The value of q_P_ in FD experiment was also higher, implying that FD experiment was more efficient.

## Discussion

The initial inoculated sludge in the two SBRs were both from an aeration tank of a WWTP in Chongqing. And both the initial concentration of MLSS and PHB in the two SBRs were the same. The only difference was whether adding a settling selection. A fast settling and withdrawal in SBR1 caused a slight more sludge discharge, therefore, the concentration of MLSS in SBR1 was slightly less than the one in SBR2 in enrichment process (Fig. 1a). A small amount of sludge with less settling ability was withdrawn at the end of feast phase through settling selection, which was assumed as the bacteria with non-capacity of storage (Chen et al. 2015). In enrichment system, bacteria was divided to two categories, bacteria with PHA accumulating ability (B1) and non-PHA accumulating bacteria (B2) (Chen et al. 2015; Jiang et al. 2012; Marang et al. 2014). The result (Fig. 1) showed that the start-up process in SBR1 (21 days) was faster than SBR2 (33 days), implying that the enrichment process just depending on metabolic selective pressure was relatively slow. The enrichment process depending on the conjunction of metabolic selective pressure and physical selective pressure was more pointed, due to the difference of the cell density at the end of feast phase (Chen et al. 2015). B1 would be heavier at the end of the feast phase, due to more PHA stored in the cells. Through a fast settling and withdrawal, B2 was weeded out. In addition, the result showed that omitting the settling selection had no obvious effect on the SBR performance. The nature of selection was to weed B2 out and maintain B1 growth. And the main goal of steady state was to maintain B1 growth and avoid B2 existing. After omitting the settling selection, the double growth limitation (DGL) strategy was still able to achieve this goal and maintain the system steady running. Therefore, the SBR performance was not altered after the omitting of the settling selection under stable operation.

The t_Fe_/t_Fa_ ratio (the length of feast time divided by the length of famine time) was a parameter in ADF strategy, considered that lower t_Fe_/t_Fa_ ratio (< 0.2–0.3) was beneficial for the enrichment (Albuquerque et al. 2010; Dionisi et al. 2006). PHA-storing bacterium had a competitive advantage under the condition of a short feast phase. On the contrary, in a longer feast phase the non-PHA-storing bacterium survived and then dominated thus resulted in the system deterioration (Dionisi et al. 2006). The t_Fe_/t_Fa_ ratio in SBR experiment and FD experiment of this study were close, about 0.46 (Table 2), higher than the range of conventional ADF but similar to the one reported by Oliveira et al. (2017). The result indicated that the befitting t_Fe_/t_Fa_ ratio of this study exceeded the range of conventional ADF strategy, hence the range of conventional ADF strategy was not suitable for estimating this SBR. The result implied that this SBR can tolerate longer feast phase and shorter famine phase but not caused the SBR collapse. On the hand, higher t_Fe_/t_Fa_ ratio was determined by the strategy of double growth limitation. In conventional ADF strategy, growth and storage process both happened after carbon source and nutrient were fed simultaneously at the initial feast phase. Differing to the aforementioned strategy, double growth limitation strategy, carbon source and nutrient fed separately, was applied in this study. Therefore, during feast phase the external carbon source was just converted to the storage polymers, implying more time needed to consume the substrate. On the other hand, higher t_Fe_/t_Fa_ ratio was determined by the goal of the coupling. In typical three-steps PHA production, the aiming of the second step was to enrich PHA-storing culture. Relative lower substrate concentration can satisfy the enrichment and growth. And substrate concentration was usually lower than 2 gCOD/L/day in conventional ADF strategy (Chen et al. 2015). In this study, to couple the enrichment and accumulation step, and harvest more PHA, more external substrate concentration was fed into the SBR, about 7.8 gCOD/L/day. More substrate caused longer storage time, therefore, higher t_Fe_/t_Fa_ ratio happened in this SBR.

To simplify the PHB production steps and decrease the cost, the enrichment and accumulation step were coupled in a SBR. The reasons for the feasibility of the coupling in this study were listed. On the hand, double growth limitation strategy was the root for the success of the coupling. In conventional ADF strategy, carbon source and nutrient were fed simultaneously at the initial feast phase, inevitably, the fate of carbon source was used to growth and storage. In this study, double growth limitation strategy ensured that storage occurred in feast phase and growth depending on PHB happened in famine phase. This is to say, carbon source was just converted to storage polymers in feast phase, meaning higher storage yield. In addition, to harvest more PHA, more external substrate concentration was fed into the SBR. Obviously, longer feast time was needed to convert external substrate to PHB. As the above paragraph description, adopting double growth limitation strategy in this study, the SBR can tolerate relative higher t_Fe_/t_Fa_ ratio and not caused the SBR collapse. On the other hand, to assure the SBR steady running, the total amount of substrate was controlled. High organic loading was considered to extend the feast length, thus non-PHA-storing culture survived and dominated (Dionisi et al. 2006). And under long-term running of high organic loading, the stability of selection by mixed culture was not maintained (Chen et al. 2017; Janarthanan et al. 2016). From the report of results section, the storage length in batch experiment for PHB storage capacity was almost 10 h in this study, not benefiting for the system stability. Therefore, the amount of Ac was controlled to insure the feast length accepted by system and adequate famine length for growth. Finally, feed-on-demand control was applied to avoid the effect of substrate inhibition. To improve the PHB production, Marang et al. (2016) increased the volume exchange ratio from 75 to 83%, however, higher volume exchange ratio lowered the operational stability. Pulsed feeding strongly improved the PHA storage efficiency in cell due to avoiding the effect of substrate inhibition (Serafim et al. 2004). From the view of substrate inhibition, the result of this study showed that PHB content was improved from 70 to 83% and the SBR stayed stable running by the application of feed-on-demand control.

In conclusions, this study demonstrates that the enrichment and accumulation step can be successfully coupled in a SBR. The settling selection-double growth limitation (SS-DGL) accelerated the start-up process. And the feeding regime of feed-on-demand (FD) in the SBR of the coupling was more favorable to PHB production. Therefore, the SS-DGL/FD-DGL strategy operated with acetate as sole carbon source supplied a fast start-up and efficient PHB production in a SBR of coupling enrichment and accumulation step.

## Additional file

**Additional file 1: Fig. S1.** The change of feeding regime from start-up period to operational period.

## Abbreviations

PHAs: polyhydroxyalkanoates
PHB: poly-β-hydroxybutyrate
SBR: sequencing batch reactor
VFAs: volatile fatty acids
ADF: aerobic dynamic feeding
ADD: aerobic dynamic discharge
SS-DGL: settling selection-double growth limitation
OUR: oxygen uptake rate
FD-DGL: feed-on-demand control-double growth limitation
COD: chemical oxygen demand
MLSS: mixed liquor suspended solids
MLVSS: mixed liquor volatile suspended solids
NH4+-N: ammonium
HAc: acetic acid

## References

[CR1] Albuquerque MG, Eiroa M, Torres C, Nunes BR, Reis MA (2007). Strategies for the development of a side stream process for polyhydroxyalkanoate (PHA) production from sugar cane molasses. J Biotechnol.

[CR2] Albuquerque MG, Torres CA, Reis MA (2010). Polyhydroxyalkanoate (PHA) production by a mixed microbial culture using sugar molasses: effect of the influent substrate concentration on culture selection. Water Res.

[CR3] Albuquerque MG, Martino V, Pollet E, Averous L, Reis MA (2011). Mixed culture polyhydroxyalkanoate (PHA) production from volatile fatty acid (VFA)-rich streams: effect of substrate composition and feeding regime on PHA productivity, composition and properties. J Biotechnol.

[CR4] Albuquerque MG, Carvalho G, Kragelund C, Silva AF, Barreto Crespo MT, Reis MA, Nielsen PH (2013). Link between microbial composition and carbon substrate-uptake preferences in a PHA-storing community. ISME J.

[CR5] Amulya K, Jukuri S, Mohan SV (2015). Sustainable multistage process for enhanced productivity of bioplastics from waste remediation through aerobic dynamic feeding strategy: process integration for up-scaling. Bioresour Technol.

[CR6] APHA (1998). Standard methods for the examination of water and wastewater.

[CR7] Beuna JJ, Dircks K, Loosdrecht MCMV, Heijnena JJ (2002). Poly-β-hydroxybutyrate metabolism in dynamically fed mixed microbial cultures. Water Res.

[CR01] Campanari S, Augelletti F, Rossetti S, Sciubba F, Villano M, Majone M (2017). Enhancing a multi-stage process for olive oil mill wastewater valorization towards polyhydroxyalkanoates and biogas production. Chem Eng J.

[CR8] Canelhas MR, Andersson M, Eiler A, Lindstrom ES, Bertilsson S (2017). Influence of pulsed and continuous substrate inputs on freshwater bacterial community composition and functioning in bioreactors. Environ Microbiol.

[CR9] Chen H, Meng H, Nie Z, Zhang M (2013). Polyhydroxyalkanoate production from fermented volatile fatty acids: effect of pH and feeding regimes. Bioresour Technol.

[CR10] Chen Z, Guo Z, Wen Q, Huang L, Bakke R, Du M (2015). A new method for polyhydroxyalkanoate (PHA) accumulating bacteria selection under physical selective pressure. Int J Biol Macromol.

[CR11] Chen Z, Guo Z, Wen Q, Huang L, Bakke R, Du M (2016). Modeling polyhydroxyalkanoate (PHA) production in a newly developed aerobic dynamic discharge (ADD) culture enrichment process. Chem Eng J.

[CR12] Chen Z, Huang L, Wen Q, Zhang H, Guo Z (2017). Effects of sludge retention time, carbon and initial biomass concentrations on selection process: from activated sludge to polyhydroxyalkanoate accumulating cultures. J Environ Sci (China).

[CR13] Chua ASM, Takabatake H, Satoh H, Mino T (2003). Production of polyhydroxyalkanoates (PHA) by activated sludge treating municipal wastewater: effect of pH, sludge retention time (SRT), and acetate concentration in influent. Water Res.

[CR14] DeSantis TZ, Hugenholtz P, Larsen N, Rojas M, Brodie EL, Keller K, Huber T, Dalevi D, Hu P, Andersen GL (2006). Greengenes, a chimera-checked 16S rRNA gene database and workbench compatible with ARB. Appl Environ Microbiol.

[CR15] Dionisi D, Majone M, Papa V, Beccari M (2004). Biodegradable polymers from organic acids by using activated sludge enriched by aerobic periodic feeding. Biotechnol Bioeng.

[CR16] Dionisi D, Majone M, Vallini G, Di Gregorio S, Beccari M (2006). Effect of the applied organic load rate on biodegradable polymer production by mixed microbial cultures in a sequencing batch reactor. Biotechnol Bioeng.

[CR17] Dionisi D, Majone M, Vallini G, Gregorio SD, Beccari M (2007). Effect of the length of the cycle on biodegradable polymer production and microbial community selection in a sequencing batch reactor. Biotechnol Progr.

[CR18] Fradinho JC, Oehmen A, Reis MA (2014). Photosynthetic mixed culture polyhydroxyalkanoate (PHA) production from individual and mixed volatile fatty acids (VFAs): substrate preferences and co-substrate uptake. J Biotechnol.

[CR19] Huang L, Chen Z, Wen Q, Lee DJ (2017). Enhanced polyhydroxyalkanoate production by mixed microbial culture with extended cultivation strategy. Bioresour Technol.

[CR20] Janarthanan OM, Laycock B, Montano-Herrera L, Lu Y, Arcos-Hernandez MV, Werker A, Pratt S (2016). Fluxes in PHA-storing microbial communities during enrichment and biopolymer accumulation processes. N Biotechnol.

[CR21] Jiang Y, Marang L, Kleerebezem R, Muyzer G, van Loosdrecht MC (2011). Effect of temperature and cycle length on microbial competition in PHB-producing sequencing batch reactor. ISME J.

[CR22] Jiang Y, Marang L, Tamis J, van Loosdrecht MC, Dijkman H, Kleerebezem R (2012). Waste to resource: converting paper mill wastewater to bioplastic. Water Res.

[CR23] Johnson K, Jiang Y, Kleerebezem R, Muyzer G, van Loosdrecht MCM (2009). Enrichment of a mixed bacterial culture with a high polyhydroxyalkanoate storage capacity. Biomacromolecules.

[CR24] Khanna S, Srivastava AK (2005). Recent advances in microbial polyhydroxyalkanoates. Process Biochem.

[CR25] Kourmentza C, Kornaros M (2016). Biotransformation of volatile fatty acids to polyhydroxyalkanoates by employing mixed microbial consortia: the effect of pH and carbon source. Bioresour Technol.

[CR26] Laycocka B, Halley P, Pratt S, Werkerc A, Lanta P (2014). The chemomechanical properties of microbial polyhydroxyalkanoates. Prog Polym Sci.

[CR27] Lemos PC, Levantesi C, Serafim LS, Rossetti S, Reis MAM, Tandoi V (2008). Microbial characterisation of polyhydroxyalkanoates storing populations selected under different operating conditions using a cellsorting RT-PCR approach. Appl Microbiol Biotechnol.

[CR28] Lu P, Zhang D, Zhang X, Cao H (2006). An implementation of the hybrid respirometric measurement principle with more reliable oxygen uptake rate (OUR) measurement. Water Pract Technol.

[CR29] Marang L, Jiang Y, van Loosdrecht MC, Kleerebezem R (2013). Butyrate as preferred substrate for polyhydroxybutyrate production. Bioresour Technol.

[CR30] Marang L, Jiang Y, van Loosdrecht MC, Kleerebezem R (2014). Impact of non-storing biomass on PHA production: an enrichment culture on acetate and methanol. Int J Biol Macromol.

[CR31] Marang L, van Loosdrecht MC, Kleerebezem R (2016). Combining the enrichment and accumulation step in non-axenic PHA production: cultivation of *plasticicumulans acidivorans* at high volume exchange ratios. J Biotechnol.

[CR32] Oehmen A, Keller-Lehmann B, Zeng RJ, Yuan Z, Keller J (2005). Optimisation of poly-β-hydroxyalkanoate analysis using gas chromatography for enhanced biological phosphorus removal systems. J Chromatogr A.

[CR33] Oliveira CSS, Silva CE, Carvalho G, Reis MA (2017). Strategies for efficiently selecting PHA producing mixed microbial cultures using complex feedstocks: feast and famine regime and uncoupled carbon and nitrogen availabilities. N Biotechnol.

[CR34] Prieto A, Escapa IF, Martinez V, Dinjaski N, Herencias C, de la Pena F, Tarazona N, Revelles O (2016). A holistic view of polyhydroxyalkanoate metabolism in *Pseudomonas putida*. Environ Microbiol.

[CR35] Reddy MV, Mawatari Y, Onodera R, Nakamura Y, Yajima Y, Chang YC (2017). Polyhydroxyalkanoates (PHA) production from synthetic waste using *Pseudomonas pseudoflava*: PHA synthase enzyme activity analysis from *P. pseudoflava* and *P. palleronii*. Bioresour Technol.

[CR36] Reis MA, Serafim LS, Lemos PC, Ramos AM, Aguiar FR, Van Loosdrecht MC (2003). Production of polyhydroxyalkanoates by mixed microbial cultures. Bioproc Biosyst Eng.

[CR37] Salehizadeh H, Van Loosdrecht MCM (2004). Production of polyhydroxyalkanoates by mixed culture: recent trends and biotechnological importance. Biotechnol Adv.

[CR38] Serafim LS, Lemos PC, Oliveira R, Reis MA (2004). Optimization of polyhydroxybutyrate production by mixed cultures submitted to aerobic dynamic feeding conditions. Biotechnol Bioeng.

[CR39] Serafim LS, Lemos PC, Albuquerque MG, Reis MA (2008). Strategies for PHA production by mixed cultures and renewable waste materials. Appl Microbiol Biot.

[CR40] Serafim LS, Lemos PC, Torres C, Reis MAM, Ramos AM (2008). The influence of process parameters on the characteristics of polyhydroxyalkanoates produced by mixed cultures. Macromol Biosci.

[CR41] Silva JA, Tobella LM, Becerra J, Godoy F, Martínez MA (2007). Biosynthesis of poly-beta-hydroxyalkanoate by *Brevundimonas vesicularis* LMG P-23615 and *Sphingopyxis macrogoltabida* LMG 17324 using acid-hydrolyzed sawdust as carbon source. J Biosci Bioeng.

[CR42] Silva F, Campanari S, Matteo S, Valentino F, Majone M, Villano M (2017). Impact of nitrogen feeding regulation on polyhydroxyalkanoates production by mixed microbial cultures. N Biotechnol.

[CR43] Solaiman DK, Ashby RD, Foglia TA, Marmer WN (2006). Conversion of agricultural feedstock and coproducts into poly(hydroxyalkanoates). Appl Microbiol Biot.

[CR44] Sudesh K, Bhubalan K, Chuah JA, Kek YK, Kamilah H, Sridewi N, Lee YF (2011). Synthesis of polyhydroxyalkanoate from palm oil and some new applications. Appl Microbiol Biot.

[CR45] Tan G, Chen C, Li L, Ge L, Wang L, Razaad I, Li Y, Zhao L, Mo Y, Wang J (2014). Start a research on biopolymer polyhydroxyalkanoate (PHA): a review. Polymers.

[CR46] Valentino F, Karabegovic L, Majone M, Morgan-Sagastume F, Werker A (2015). Polyhydroxyalkanoate (PHA) storage within a mixed-culture biomass with simultaneous growth as a function of accumulation substrate nitrogen and phosphorus levels. Water Res.

[CR47] Wang X, Oehmen A, Freitas EB, Carvalho G, Reis MA (2017). The link of feast-phase dissolved oxygen (DO) with substrate competition and microbial selection in PHA production. Water Res.

[CR48] Werker AG, Bengtsson SOH, Karlsson CAB (2014) Method for accumulation of polyhydroxyalkanoates in biomass with on-line monitoring for feed rate control and process termination, Vol. US 8,748,738 B2, Veolia Water Solutions and Technologies, Saint-Maurice (FR)

[CR49] Yuan H, Chen Y, Zhang H, Jiang S, Zhou Q, Gu G (2006). Improved bioproduction of short-chain fatty acids (SCFAs) from excess sludge under alkaline conditions. Environ Sci Technol.

