# Supplementary material for: Improving PHA production in a SBR of coupling PHA-storing microorganism enrichment and PHA accumulation by feed-on-demand control
Source: AMB Express. 2018 Jun 12;8:97. doi: 10.1186/s13568-018-0628-x (PMC5997608; doi:10.1186/s13568-018-0628-x)
Supplement: Supplementary file 1 — Additional file 1: Fig. S1. The change of feeding regime from start-up period to operational period. [file 13568_2018_628_MOESM1_ESM.pdf]

Improving PHA production in a SBR of coupling PHA-storing microorganism enrichment and PHA accumulation by feed-on-demand control

Shanwen Zeng [1,2], Fuzhong Song [1,2,*], Peili Lu [1,2], Qiang He[3,4], Daijun Zhang [1,2,*]

1. State Key Laboratory of Coal Mine Disaster Dynamics and Control, Chongqing University, Chongqing 400044, China;

2. Department of Environmental Science, Chongqing University, Chongqing 400044, China;

3. Faculty of Urban Construction and Environmental Engineering, Chongqing University, Chongqing, 400044, China;

4. Key Laboratory of the Three Gorges Reservoir's Eco-Environments, Ministry of Education, Chongqing University, Chongqing, 400044, China.

Corresponding author: F. Song & D. Zhang

Tel: +86 023 65105875

Fax: +86 023 65105875.

E-mail: cqsfz@cqu.edu.cn (F. Song) & dzhang@cqu.edu.cn (D. Zhang)

*Corresponding author. Address: Department of Environmental Science, Chongqing University, Chongqing 400044, China. Tel.: +86-023-65105875; fax: +86-023-65105875.
E-mail address: cqsfz@cqu.edu.cn (F. Song) & dzhang@cqu.edu.cn (D. Zhang)

S1 The change of feeding regime from start-up period to operational period

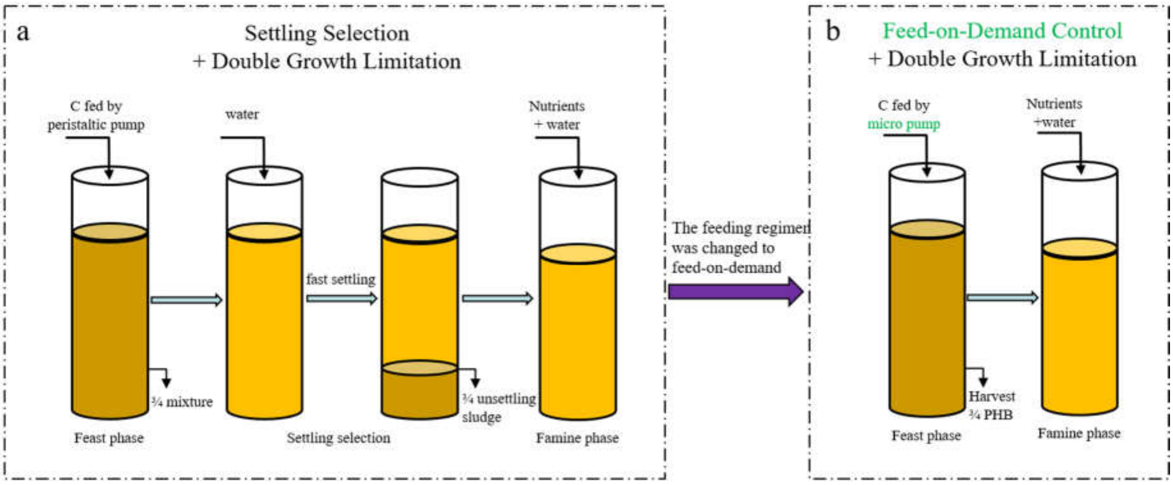

Fig. S1 PHA production in a SBR of coupling the enrichment and accumulation, (a)Start-up, (b) Operational period.
